# Synthesis of current pediatric urinary microbiome research

**DOI:** 10.3389/fped.2024.1396408

**Published:** 2024-06-18

**Authors:** Layla M. Jeries, Tatyana A. Sysoeva, Lisa Karstens, Maryellen S. Kelly

**Affiliations:** ^1^ Department of Biological Sciences, The University of Alabama in Huntsville, Huntsville, AL, United States; ^2^ Department of Medical Informatics and Clinical Epidemiology, Oregon Health & Science University, Portland, OR, United States; ^3^ Department of Obstetrics and Gynecology, Oregon Health & Science University, Portland, OR, United States; ^4^ Division of Healthcare of Women and Children, School of Nursing, Duke University, Durham, NC, United States; ^5^ Department of Urology, Duke University Hospital, Durham, NC, United States

**Keywords:** urinary microbiome, urobiome, pediatric urobiome, pediatric bladder microbiome, urinary mycobiome, urinary virome

## Abstract

The human urinary bladder hosts a complex microbial community of low biomass referred to as the urobiome. While the composition of the urobiome has been investigated in adults for over a decade now, only a few studies have considered the presence and composition of the urobiome in children. It is critical to explore how the urobiome develops throughout the life span and how it changes in the presence of various health conditions. Therefore, we set to review the available data on pediatric urobiome composition and its development with age and disease. In addition, we focused on identifying and reporting specific gaps in our knowledge of the pediatric urobiome that we hope will be addressed by future studies in this swiftly developing field with fast-improving methods and consensus.

## 1 Introduction

The urinary microbiome, or urobiome, comprises the microbial community of the urinary tract, which includes the kidneys, ureters, bladder, and urethra. Standard urine culture (SUC) long supported the paradigm that urine and the urinary tract were sterile except in the case of infection (1). However, the development of expanded quantitative urine culture (EQUC) and culture-independent 16S rRNA gene sequencing have allowed the detection and characterization of primarily bacteria from urine samples (2, 3). As with other microbial niches of the human body, the urobiome has been suggested to contribute to both urinary health and disease. Elevated or reduced populations of microorganisms that can potentially lead to dysbiosis have been associated with urologic diseases, such as prostate cancer, bladder cancer, chronic prostatitis/chronic pelvic pain syndrome, urge urinary incontinence, overactive bladder, stone disease, and urinary tract infection (UTI) [reviewed in (4)].

The last decade has seen an increase in the study of the urobiome, examining cohorts that include one or both sexes, as well as various infectious states and pre-existing conditions. The urobiome varies greatly between individuals and is expected to be uniquely defined, yet dynamic, within an individual across time, as has been found in a study of premenopausal women (5). Several bacterial taxa can be found in the urobiomes of adult males and females, such as *Streptococcus, Staphylococcus,* and *Corynebacterium* (6–9)*.* However, certain bacteria tend to be predominant more frequently within a specific sex, such as lactobacilli, which tend to be predominant bacteria in women (10, 11) but not in men, and *Staphylococcus* which is sometimes a predominant bacterium in males (12–14), but less often in females. Importantly, the composition of the urobiome can vary by sample collection technique, as it dictates which niche bacteria are collected from. Catheter and suprapubic aspirates sample the bladder directly with little contribution from surrounding areas (2), whereas voided samples typically include bacteria from the genitals. Thus, the former are referred to as bladder microbiome and the latter as urogenital microbiome (15).

Questions remain regarding the development of the urobiome in childhood across age, sex, toilet-training status, and pubertal stage, as well as how urologic and non-urologic diseases might be affecting urobiome composition and abundance. How the urobiome develops during childhood and how this may contribute to disease states or promote health is unknown. This review aims to summarize the current data regarding urobiome composition of different pediatric cohorts. We examine the implications of these results for the health and disease of the urinary tract and discuss how the pediatric cohort influences study design. Finally, we highlight the next steps necessary to further discern the dynamics of the pediatric urobiome.

## 2 Methods employed in pediatric urobiome studies

**Figure 1:**
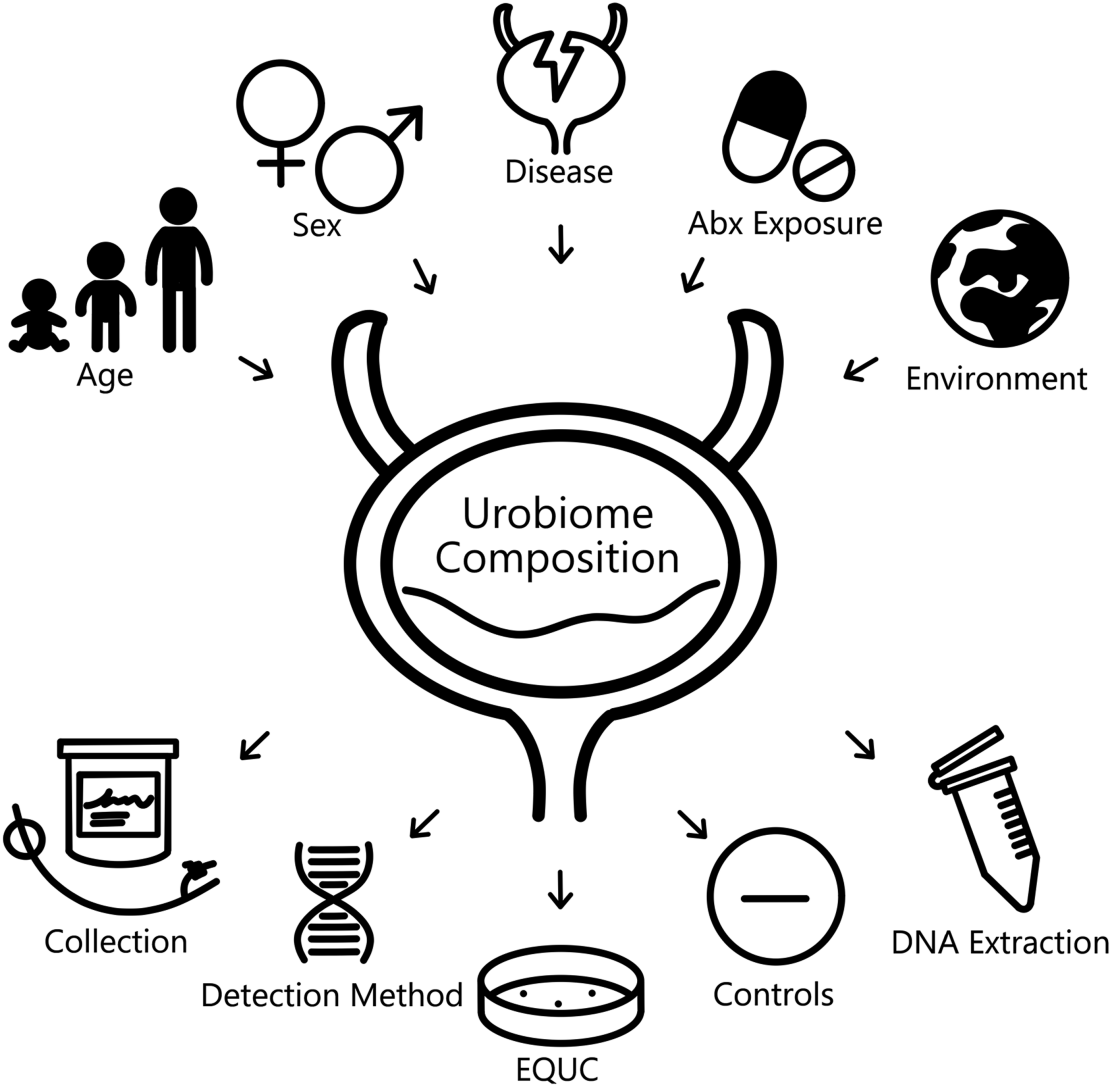
Summary of variables affecting observed urobiome composition.

Several reviews have summarized important considerations for designing and understanding urobiome studies (15–17). There are numerous variables and factors of interest that can affect urobiome composition and abundance (Figure 1). For example, differences in the urobiome based on sex and age have been observed in adult cohorts, and this question persists in pediatric cohorts, especially in regard to development from birth into childhood, adolescence, and young adulthood. It is also important to consider whether disease, antibiotic exposure, and other environmental/regional factors have influence. There is also the question of whether collection methods and downstream processing of samples can further determine the resolution and accuracy with which these measures are detected. Specific features of pediatric cohorts limit the use of some techniques and require additional considerations. Herein, we highlight those unique features in the context of typical urobiome study and identify current trends in pediatric urobiome study design.

### 2.1 Sample collection

Combining the nature of urine being a low biomass sample (15, 16) with the smaller bladder capacity of children compared to adults means sample collection must be carefully considered in pediatric cohorts. The two most common techniques for obtaining urine samples in pediatric and adult urobiome studies are clean-catch midstream urine (CCMSU) and transurethral catheterization (TUC). Suprapubic aspiration is another sampling method in adult urobiome studies and is considered to be the “cleanest” method of sampling (2), but it has not yet been employed in pediatric cohorts due to its high degree of invasiveness and ethical considerations. Across studies presented in this review (Table 1), there is an almost equal share of either CCMSU or TUC collection methods used to sample the urobiome.

**Table 1:** Summary of the highlighted methods employed across studies of the pediatric urobiome.

Study	Collection method	Sequencing (and hypervariable region if 16S)	OTUs or ASVs	EQUC
Healthy cohorts				
Fredsgaard et al. (18)	CCMSU	16S: V4	OTU	
Storm et al. (19)	TUC and perineal and distal urethral swabs	16S: V4	ASV	X
Kassiri et al. (20)	TUC	16S: V6	OTU	
Reasoner et al. (21)	TUC	16S: V4	ASV	X
Wehedy et al. (22)	CCMSU	Shotgun metagenomics	OTU	
Neurogenic bladder				
Forster et al. (23)	TUC	16S: V4	OTU	
De Maio et al. (24)	CCMSU	16S: V5–V6	ASV	
Vesicoureteral reflux				
Vitko et al. (25)	TUC, CCMSU for controls	16S: V4	OTU	
Kelly et al. (26)	TUC	16S: full length	ASV	
Bladder and bowel dysfunction				
Cole et al. (27)	CCMSU	16S: V4	ASV	X
Urinary tract infection				
Kinneman et al. (28)	TUC	16S: V3–V4	OTU	
Forster et al. (29)	TUC	ITS2	ASV	
Mental disorder				
Cho et al. (30)	CCMSU	16S: V3-V4	OTU	

CCMSU, clean-catch midstream urine; TUC, transurethral catheterization; OTU, operational taxonomic unit; ASV, amplicon sequence variant; EQUC, expanded quantitative urine culture.

CCMSU is the least invasive method of collecting urobiome samples but it is prone to collecting some microbes from the surrounding urogenital microbiomes, which Storm and colleagues observed to differ from urethral urobiomes (19). Possible skin and adjacent niche contributions, such as from the vagina and anus, can be accounted for via swabbing and culturing or sequencing of the surrounding urogenital regions. Another limiting factor of the CCMSU method is that children must be able to volitionally void. So, this method potentially excludes younger children and infants whose samples could provide greater insight into the acquisition and early-life development of the urobiome. Similarly, younger children also require assistance providing samples and/or can potentially contaminate samples. While, ultimately, CCMSU contains what is more appropriately described as a urogenital microbiome sample than of the urinary tract microbiome (15), it is often the most feasible option in pediatric cohorts.

TUC requires the insertion of a urinary catheter into the bladder. While this allows for sampling of the bladder and opens cohorts to virtually all ages, TUC is much harder to justify ethically for use in healthy children as this carries the risk of infection, inflammation and injury, and can cause undue stress and pain to a child. The studies that have employed this method take advantage of TUC already being ordered for other procedures, such as a voiding cystourethrogram, surgery, or in children who perform catheterization regularly due to urinary retention. While TUC is also prone to possible contribution from surrounding skin during catheter insertion, particularly in studies of uncircumcised males, this is reduced compared to CCMSU.

### 2.2 Sequencing methods

The available studies thus far have primarily relied on culture-independent 16S rRNA gene amplicon sequencing to identify constituents of pediatric urobiomes (Table 1). This method relies on isolating DNA, amplifying region(s) of the 16S rRNA gene of bacteria and performing high-throughput sequencing, such as on the Illumina sequencing platform. Usually, the amplified regions comprise one to three hypervariable (V) regions out of the nine total V regions identified in the bacterial 16S rRNA gene, with V1–V3 and V4–V6 being the most common ranges. Multiple reads are then processed to produce amplicon sequence variants (ASVs) or operational taxonomic units (OTUs) for downstream analysis. ASVs are generated by employing an error correction model, whereas OTUs are generated by grouping sequences that share identity above a similarity threshold, typically 97% but occasionally 99% (31–33). Benchmarking OTUs vs. ASVs has revealed significant differences in diversity metrics and identified taxa, generally favoring ASVs (34, 35). While OTUs have not been fully abandoned (36, 37), there has been a consistent shift towards the use of ASVs in the most recent pediatric urobiome studies (Table 1).

The use of the 16S rRNA gene for sequencing has several important implications. First, the taxonomic resolution of short 16S amplicons is considerably low. Most analyses distinguish down to the genus level at best, though families or higher can often be the limit. Species resolution is rare but possible for some species (38), and it cannot provide strain level distinction. With the additional factor of potential reclassification of species and genera, mapping reads to recent nomenclature changes further complicates identification (39). Additionally, the 16S rRNA gene is specific to bacteria. Therefore, 16S sequencing overlooks other microbes that might also be present in the urinary tract. Detection and classification of other microbes require applying different detection methods, such as sequencing of the internal transcribed spacer (ITS) sequence for fungi, specific 16S sequencing for archaea, or metagenomic sequencing of the entire microbial biomass including viruses.

Metagenomic sequencing allows for reading of the entire complement of DNA present in a particular niche. This type of sequencing analysis applied to urine is complicated by the low biomass of this microbial community, as well as the high and variable number of host cells and DNA present that complicate the analysis of metagenomic sequencing results. This results in a need to increase the coverage of sequencing, apply complex and expensive methods for microbial DNA enrichment, and involves intricate bioinformatics. Alternative methods that might bring higher taxonomic resolution with a lower cost than true metagenomics include the use of long-read sequencing (PacBio, Nanopore) or synthetic long-read methods that can provide data on longer regions of interest such as the entire 16S rRNA gene (40).

While it might not be feasible to apply the most advanced methods listed above to all samples due to cost or expertise, it is likely possible to run benchmarking studies to compare different methods’ outcomes to obtain an estimate on whether they are comparable and/or biased. This has been done for adult urobiome studies (41, 42). Additionally, a consensus has been released which highlights similar considerations for the study of urobiomes in general (15).

### 2.3 Expanded quantitative urine culture

Initially SUC was developed to detect major uropathogens for UTI diagnostics. SUC typically involves plating urine samples onto 5% sheep blood agar and MacConkey agar plates and then incubating aerobically at 35°C for 24 h. Therefore, SUC caters mostly to *Escherichia coli* and other fast-growing, facultative anaerobes with similar growth requirements. EQUC was developed to encompass a greater variety and multiple combinations of incubation times, culture media, and atmospheric environments (3, 17). Therefore, EQUC has enabled the characterization of bacteria otherwise not detected or detected less frequently when using traditional SUC technique (43–45). Unlike culture-independent sequencing methods described above, EQUC allows identification of microbes that are viable while in urine and, at the same time, for isolation and storage of those organisms for further studies. These isolates can be identified to species and strain level using matrix-assisted laser desorption ionization–time of flight (MALDI-TOF) mass spectrometry, 16S rRNA gene sequencing, and whole genome sequencing.

Only three of the identified pediatric urobiome studies use EQUC (Table 1). Storm et al. (19) employed EQUC coupled with MALDI-TOF and culture-dependent 16S sequencing for taxonomic identification. Their culture-independent 16S sequencing alone was able to detect bacteria in 84% of urine samples, and EQUC was able to detect bacteria in 60%. Combined, bacteria were detected in 91% of all urine samples. Swabs of adjacent niches had similar results (>90%). Reasoner et al. (21) modified the EQUC protocol and with its use, identified growth in 65% of urine samples in at least one growth condition. They were also able to detect additional families and species in their samples using MALDI-TOF. Cole et al. (27) similarly employed these methods and were able to verify their 16S-based taxa identification.

This way, the shift to predominance of *Lactobacillus* and *Bifidobacterium* in post-pubertal females in the study by Storm et al. (19) was observed by both EQUC and sequencing, and the composition and abundance measured by EQUC by Cole et al. (27) overall agreed with culture-independent 16S sequencing. However, EQUC and culture-independent 16S sequencing do not always reconcile. For example, the families detected most frequently by EQUC by Reasoner et al. (21) were not detected in culture-independent sequencing results at all, and several families detected most frequently by 16S sequencing were not detected in EQUC isolates. 16S sequencing methods are limited by available mapping to known genetic sequences in available databases, and EQUC still is not a fully comprehensive method of culturing all species. At their current level of development, combining EQUC and sequencing can reveal what a single method cannot, but further use and development of these techniques is necessary and can improve analyses of urobiome composition.

## 3 Summary of current studies

We have identified thirteen published studies that investigate the composition of the pediatric urobiome (Table 1). Herein, we summarize the results for each study and compare these with similar studies, both pediatric and adult. We also provide potential insight into the larger context of the connection between the urobiome and health and disease. The urobiome findings of each study are summarized in Table 2.

**Table 2:** Summary of study features and reported urobiome composition based on culture-independent sequencing.

Study	Sample size	Sex	Age	Urobiome composition
Healthy cohorts				
Fredsgaard et al. (18)	30	50% male, 50% female	6 years–10 years	Males: *Porphyromonas, Ezakiella, Campylobacter, Prevotella, Dialister* Females: *Prevotella, Porphyromonas, Ezakiella, Prevotella 6, Dialister*
Storm et al. (19)	74	65% male, 35% female	2 weeks–17.4 years	Males: *Prevotella, Staphylococcus, Corynebacterium, Streptococcus, Winkia* Females: *Lactobacillus, Bifidobacterium, Veillonella, Prevotella, Winkia, Schaalia*
Kassiri et al. (20)	5	100% male	3 months–8 years	*Staphylococcus, Varibaculum, Peptoniphilus, Actinobaculum*
Reasoner et al. (21)	50	100% male	6 months–12 months	*Nocardiopsis, Acinetobacter, Saphylococcus, Escherichia-Shigella, Pseudomonas, Lacibacter, Lactobacillus, Prevotella*
Wehedy et al. (22)	40	62.5% male, 37.5% female	1 year–17 years	Bacteriome: *Arthrobacter* sp.*, Acidothermus* sp. in males; *Anaerococcus* sp.*, Viellonella* sp*.* in females Mycobiome: *Saccharomyces* sp. in males; *Malassezia* in females Virome: Caudovirales, Tymovirales, Herpesvirales
Neurogenic bladder				
Forster et al. (23)	34	55.9% male, 44.1% female	2 months–21 years	Enterobacteriaceae, *Klebsiella, Staphylococcus, Streptococcus*, Neisseriaceae
De Maio et al. (24)	79	∼60% male, ∼40% female	1 year–18 years	*Enterococcus, Escherichia-Shigella*
Vesicoureteral reflux				
Vitko et al. (25)	49	24.5% male, 75.5% female	Undefined pediatric age range, 9 adult controls	VUR patients dominated by either *Dorea, Escherichia* Healthy controls dominated by *Prevotella, Ezakiella,* or *Lactobacillus*
Kelly et al. (26)	54	39.4% male, 60.6% female	3 months–11 years	Males: *Peptoniphilus, Ezakiella, Sphingomonas, Ralstonia,* and *Anaerococcus*. Females: *Prevotela, Peptoniphilus, Anerococcus, Ezakiella,* and *Streptococcus*
Bladder and bowel dysfunction				
Cole et al. (27)	33	100% female	Prepubertal, >2 years	*Peptoniphilus, Anaerococcus, Lactobacillus, Fenollaria, Finegoldia, Escherichia, Campylobacter, Streptococcus*
Urinary tract infection				
Kinneman et al. (28)	85	30.6% male, 69.4% female	<48 months	*Prevotella, Peptoniphilus, Escherichia, Veillonella, Finegoldia*
Forster et al. (29)	374	25.7% male, 74.3% female	1 month–3 years	Mycobiome, with UTI: *Stereum complicatum, Trametes versicolor, Candida parapsilosis, Cladospoium halotolerans-sphaerospermum, Cantharellales* spp. Mycobiome, without UTI: *Stereum complicatum, Caldosporium, Malassezia restrica, Trametes versicolor*
Mental disorder				
Cho et al. (30)	72	100% male	6 years–19 years, adult controls (19–years 23 years)	*Ralsonia, Afipia*

Sample size reflects the total number of samples that produced reads, not study enrollment, when applicable. Sex composition attempts to reflect the percentage of read-producing samples corresponding to each sex, but generally, the percentages based on the number of enrolled subjects were all that were provided. Urobiome composition focuses on the abundant genera reported by studies; when genera were not reported/highlighted, higher taxa or species were listed instead.

### 3.1 Healthy prepubertal urobiome

Several studies have examined cohorts of children that were considered “healthy”, meaning that they were asymptomatic for UTI and had urinary continence. Fredsgaard et al. (18) examined a prepubertal cohort of 30 children comprising ages 6 years to 10 years and an equal male-to-female sex ratio. Participants had not been exposed to antibiotics within the 3 months prior to CCMSU sample collection. Diversity was significantly higher in females, but there was no difference in the relative abundance of each genus. Males and females exhibited some similarities in the most abundant genera; however, the relative abundances of these genera were significantly different. *Porphyromonas* was the most abundant in males, followed by *Ezakiella, Campylobacter, Prevotella*, and *Dialister. Prevotella* was the most abundant in females, followed by *Porphyromonas, Ezakiella, Prevotella 6*, and *Dialister*.

The cohort examined by Storm et al. (19) consisted of 74 children aged 2 weeks to approximately 17 years undergoing anesthesia for various urologic/specialty procedures. Still, these children were otherwise considered healthy, had no prior urinary tract surgeries, possessed a healthy immune system, and had not been exposed to antibiotics within 3 months prior to sample collection. In addition to TUC urine samples, swabs of the adjacent microbial niches were taken, including the urethra, perineum, vagina for females, and foreskin for males. The most common genera in male urobiomes were *Prevotella, Staphylococcus, Corynebacterium, Streptococcus,* and *Winkia*. Neither composition nor diversity appeared to change with age. In prepubertal female urobiomes the most common genera were *Lactobacillus, Bifidobacterium, Veillonella, Prevotella, Winkia,* and *Schaalia*. Both composition and diversity did change with age; *Lactobacillus* and *Bifidobacterium* became predominant after puberty in the female urobiome and were similarly observed in the urethral and vaginal microbiomes. Males demonstrated reduced diversity in microbiome composition of their urogenital niches compared to females. In both sexes, diversity was observed to be reduced prior to toilet training, but this was especially evident in females.

Two studies have examined exclusively male infants and/or prepubertal males. The earliest, by Kassiri et al. (20), identified evidence of a urobiome in TUC samples of prepubertal males as young as 3 months and as old as 8 years. *Staphylococcus* and *Varibaculum* had the highest abundance, followed by *Peptoniphilus* and *Actinobaculum*. This cohort included an equal mix of those who did and did not have prior antibiotic exposure, but no statistically significant difference in alpha diversity was observed between these groups. Principal component analysis (PCoA) showed that urobiomes of the two groups clustered separately, which suggested dissimilarities between the composition of the two groups, but the especially small sample size of this study (*n* = 5) made it impossible to draw definitive conclusions.

The latest of the two studies, by Reasoner et al. (21), also examined a tighter age range of 50 prepubertal males—infants aged 6–12 months—and was the first to examine a cohort that did not contain any individuals possessing any structural or functional urinary tract abnormalities nor had experienced any prior UTI. *Nocardiopsis* and *Acinetobacter* were detected in all TUC samples, and *Staphylococcus, Escherichia-Shigella*, and *Pseudomonas* were detected in over 90% of samples. *Lacibacter, Lactobacillus*, and *Prevotella* were also abundant, though detected variably across samples. Mode of delivery and prior antibiotic exposures did not appear to influence alpha diversity or overall urobiome composition significantly.

Many genera identified in the urobiome of children match those identified in their adult counterparts. For example, *Staphylococcus* and *Corynebacterium* have now been observed in the urobiomes of both healthy adult males and healthy male children (6, 7, 20). The question of how the healthy urobiome develops in early life is of notable interest in the study of pediatric urobiomes. Interestingly, the prepubertal cohorts comprising both sexes share many abundant genera, which could suggest that later pubertal changes are an important influence in differentiating the healthy male and female urobiomes. Across studies, *Prevotella* have been observed frequently in samples, regardless of the sex or age composition of cohorts. *Prevotella* are Gram-negative, nonmotile anaerobes known to inhabit other niches such as the skin, oral cavity, vagina, and the gastrointestinal tract (46). The studies thus far have not identified strong correlations between urobiome composition or abundances and mode of delivery, unlike the pattern identified in microbiome studies of other anatomical locations (47). In early life, *Prevotella, Viellonella,* and *Clostridium* have been transiently detected at the buttock, along with more frequently detected species of *Staphylococcus*, *Streptococcus*, and members of the order Enterobacteriales (48). *Prevotella* and other abundant genera in adjacent niches could contribute to similar observations in the pediatric urobiome, especially in these cohorts that consist of pre-toilet trained children.

### 3.2 Urologic conditions

Compared to studies of healthy children, there has been more work that has gone into the pediatric urobiome’s potential relationship with urinary tract pathologies, including neurogenic bladder (NB), vesicoureteral reflux (VUR), bladder and bowel dysfunction (BBD), and UTI. These studies have identified potential differences in genera composition and relative abundances compared to those observed in the urobiomes of healthy individuals.

#### 3.2.1 Neurogenic bladder

Several adult studies have identified distinct variations in the urobiomes of individuals with NB compared to those of healthy individuals. Notably, most genera identified at the highest abundance contain many known uropathogens, including *Enterococcus, Escherichia, Klebsiella,* and *Pseudomonas* (49–51)*.* Additionally, lactobacilli have been observed to be greatly reduced in adult female neurogenic urobiomes compared to healthy females (51).

Similar genera have been observed in the urobiomes of children with NB, and their overall urobiome composition appears to have varying and overall reduced abundances of identified genera compared to healthy children. Forster et al. (23) aimed to investigate the baseline urobiome of 34 children—aged 2 months to 21 years—with NB and determine compositional differences among those with negative SUC, asymptomatic bacteriuria (ASB), or UTI. The family Enterobacteriaceae (unspecified genera) were the most abundant across all TUC samples, followed by genera *Klebsiella*, *Staphylococcus*, *Streptococcus*, and family Neisseriaceae (unspecified genera). There were too few participants to statistically compare the composition between those who had a Mitrofanoff, had both a Mitrofanoff and an augmented bladder, or neither. There were no statistically significant differences in composition or diversity within samples between participants with negative SUC, ASB, or UTI.

De Maio et al. (24) analyzed CCMSU samples from 79 children aged 1–18 years and found that those with spina bifida (SB) possessed an altered urobiome compared to healthy controls. The phyla Actinobacteria, Bacteroidetes, Firmicutes, and Proteobacteria were detected in both groups; compared to healthy controls, the phylum Proteobacteria was detected in higher abundance, and the phyla Bacteroidetes and Firmicutes were in lower abundance. Genera associated with UTI, such as *Enterococcus* and *Escherichia-Shigella,* were also more abundant in the SB samples compared to healthy controls. There were no statistically significant differences in alpha diversity, but beta diversity did differ between the two groups; PCoA also reiterated this difference. Clean intermittent catheterization (CIC) usage also significantly affected abundance of some taxa. The phylum Bacteroidetes, which was reduced in individuals with SB compared to healthy controls, was further reduced in those with SB who performed CIC*.* Additionally, several genera *Faecalibacterium, Lactobacillus, Staphylococcus*, and *Streptococcus* were significantly reduced in those with SB who performed CIC.

Two additional studies have examined the urobiome of children with NB in the context of urobiome intervention and alteration. Because these are intervention studies and not observational of baseline urobiome dynamics, we only briefly highlight their results in the context of NB. Kispal et al. (52) observed a high abundance of *Corynebacterium* followed by *Pseudoxanthomonas, Lactobacillus, Flavobacterium*, and *Micrococcus* post-bladder augmentation with either colon or ileum mucosal tissue across 4 years of observation, regardless of the origin of the mucosal tissues used. Forster et al. (23) administered intravesical instillations of the common probiotic *Lactobacillus rhamnosus* GG [now reclassified as *Lacticaseibacillus rhamnosus* (39)] into neurogenic bladders. While initial post-instillation urinary symptoms arose in some children, it was overall well-tolerated, and *Lactobacillus* was successfully introduced and/or maintained in most. While both studies had small sample sizes, they demonstrate the promise and potential next steps in understanding how the urobiome modulation can be harnessed to treat urinary tract disease.

#### 3.2.2 Vesicoureteral reflux

There have been two studies examining the urobiome in children with VUR. Vitko et al. (25) were the first to report a baseline urobiome profile in VUR patients. They examined a cohort of 49 individuals that consisted of healthy controls and VUR patients, either lacking or accompanied by renal scarring. This cohort was notably a broader, undefined pediatric range that did include 9 adult controls. Regardless of scarring, VUR participants exhibited a predominance of *Dorea* and/or *Escherichia* in TUC samples. Healthy controls possessed urobiomes dominated by either *Prevotella*, *Ezakiella*, or *Lactobacillus*. Adjusting for age, sex, and antibiotic usage, composition differed significantly among their participant categories. While some healthy controls did include adult CCMSU samples, the predominant genera of the reported healthy urobiomes matched those detected in abundance in the identified healthy pediatric urobiomes. Meanwhile, the urobiome of children with VUR was dominated by the genera associated with infection, further supporting the idea that the urobiome is tied to urinary tract health and disease.

Kelly et al. (26) examined the urobiome in samples of 54 children aged 3 months to 10.8 years that were referred for a voiding cystourethrogram (VCUG) due to urologic concerns. This study took advantage of TUC samples obtained during the procedure and used synthetic long-read sequencing of the entire 16S rRNA gene for improved taxonomic resolution of the urobiome composition. The majority of subjects were either confirmed to possess VUR or had a history of VUR that resolved, but none utilized intermittent catheterization or had a diagnosis of neurogenic bladder. Male urobiomes were observed to be dominated by *Peptoniphilus, Ezakiella, Sphingomonas, Ralstonia*, and *Anaerococcus.* Female urobiomes were dominated by *Prevotela, Peptoniphilus, Anerococcus*, *Ezakiella*, and *Streptococcus*; *Anaerococcus* and *Prevotella* were in significantly higher abundance in females compared to males. Females exhibited overall greater diversity than males, but this diversity was relatively consistent across ages in females and increased with age in males.

#### 3.2.3 Bladder and bowel dysfunction

Cole et al. (27) sought to compare the urogenital microbiome of 33 healthy children with that of children with BBD. The study population consisted of 33 prepubertal females over the age of 2 years who had completed toilet training at least 6 months prior to collection, which is especially important in the context of the chosen CCMSU collection method. Both healthy controls and those with BBD had microbiomes consisting of shared core genera: *Peptoniphilus, Anaerococcus, Lactobacillus, Fenollaria,* and *Finegoldia.* However, several genera predominantly observed in the BBD group were not observed in the healthy controls, including *Escherichia, Campylobacter,* and *Streptococcus*. These genera contain many species found to cause opportunistic infection, although the study exclusion criteria had prevented those with symptomatic infection from enrolling. However, overall alpha and beta diversity did not differ significantly between the healthy and BBD groups, and the authors concluded that the urogenital microbiomes did not differ significantly.

#### 3.2.4 Urinary tract infection

The connection between the urobiome and UTI/recurrent UTI in adults is being elucidated (17, 45, 53–56). Current work has reported distinct urobiome profiles when comparing healthy and infected patients; as similarly noted in our previous discussions, pathogenic genera tend to predominate in UTI, including *Klebsiella*, *Pseudomonas*, *Enterobacter,* and *Enterococcus* (57, 58)*.* Herein we specifically highlight bacterial composition and abundances; we continue this discussion with the inclusion of fungi in the following section.

Pathogenic genera have also been identified at higher abundances in pediatric urobiome samples that were positive for UTI. Kinneman et al. (28) examined a cohort of 85 children under 2 years of age who were suspected to have a UTI, with most being febrile; they otherwise exhibited no urinary tract pathologies or complications. The study was able to identify the most abundant urobiome members of different taxonomic levels, including phyla, classes, orders, families, and genera. However, the latter is what we choose to highlight exclusively here for the sake of more closely comparing it with other studies. Identified genera overall included *Prevotella, Peptoniphilus, Escherichia, Veillonella,* and *Finegoldia*. The nine participants that were indeed diagnosed with UTI all exhibited an increased abundance of *Escherichia/Shigella*. Alpha diversity was significantly reduced in those with a confirmed UTI or symptom of hematuria. The use of antibiotics within the 2 weeks prior to TUC sample collection significantly affected alpha and beta diversity. Other demographics (including sex) and mode of delivery did not appear to affect either diversity measure. Outside the context of UTI, healthy results align with some of the abundant genera observed in urobiomes of the previously discussed healthy pediatric cohorts, including *Prevotella, Veillonella*, and *Peptoniphilus*. The results from the small sample of individuals diagnosed with a UTI aligned with prior works identifying reduced diversity and a predominance of genera typically associated with infection.

Similarly, the previously discussed study by Kelly et al. (26) also examined differences in composition between groups of children who had a history of one, two, or three or more UTIs. Those with three or more UTIs had significantly reduced diversity when compared with those with one UTI. Overall abundances of certain taxa were observed to be significantly decreased in those with 3 or more UTIs, such as *Lawsonella, Corynebacterium,* and, interestingly, *Enterococcus,* which have been previously been described to be predominate in UTI*.* This cohort did include children who had been treated with antibiotics prior to enrollment or were concurrently taking prophylactic antibiotics, which may have contributed to the observed differences in urobiome features, but overall, there was no significant difference between the two groups of exposure.

### 3.3 Metagenomics and urinary mycobiomes/viromes

Other human microbiome niches have been shown to contain microbes other than bacteria, such as archaea, fungi, protists, and viruses, including bacteriophages (59). There have been several studies that have expanded the analysis of the urobiome of adults to the characterization of present fungi and viruses (57, 60–64). Recently, two studies—Wehedy et al. (22) and Forster et al. (29)—have similarly applied metagenomic and ITS sequencing methods to pediatric cohorts.

Wehedy et al. (22) were the first to use metagenomic sequencing to characterize children's true urinary profile containing the urinary bacteriome, mycobiome, and virome. They examined 40 healthy children, primarily comparing by sex and age (1–5 years, 6–10 years, and 10–18 years). Eight nationalities were included in this study, but no significant differences were observed among them. All participants were considered healthy and had not been exposed to antibiotics within 2 months prior to CCMSU sample collection.

Bacteria were the most abundant domain detected. Differences in beta diversity were significant between males and females. Firmicutes, Actinobacteria, and Proteobacteria dominated detected phyla in both sexes, with Firmicutes were significantly higher in females and Actinobacteria were higher in males. Genera *Anaerococcus* and *Viellonella* were significantly higher in females, and genera *Arthrobacter* and *Acidothermus* were significantly higher in males. Metagenomic analysis allowed for species-level resolution, revealing higher abundances of *Anaerococcus pervotii, Anerococcus vaginalis,* and *Viellonella parvula* in females, and higher abundances of *Arthrobacter* spp., *Arthrobacter aurescens*, *Thermobifida fusca*, and *Acidothermus cellulolyticus* in males. Across age groups, there were no statistically significant differences in alpha or beta diversity, and there were no significant differences in genera and species composition.

The urinary mycobiomes, or uromycobiomes, of this cohort were dominated by phylum Ascomycota in males and Basidiomycota in females. At the species level, *Saccharomyces pastorianus* and *Saccharomyces cerevisiae* were highest in males, and *Malassezia lobose* was highest in females. Unlike the results observed in bacterial abundances, the two sexes had no significant difference in beta diversity. Age groups also saw no significant diversity differences, except for a significant difference in beta diversity between the 6–10 years and >10 years age groups.

Using sequencing of the ITS region for fungi, Forster et al. (29), also detected uromycobiomes in the largest pediatric cohort examined in a urobiome study to date: 50 prepubertal children (aged 1 month to 3 years) with UTI and 324 without UTI. Both groups shared *Stereum complicatum* and *Trametes versicolor* within their top four most abundant fungal species. *Candida parapsilosis* and *Cladosporium halotolerans-sphaerospermum* were the other two most detected species in TUC samples of those with UTI, and *Malassezia restricta* and *Cladosporium* spp. were the other two most detected in those without UTI. Additionally, *Cantharellales* spp. were detected exclusively in those with UTI. Younger children and those with UTI were more likely to have detectable mycobiomes, but there was no difference based on sex. Overall, those with UTI had higher total fungal cell counts compared to those without. Interestingly, alpha diversity of fungi was higher in those with UTI than those without, but no difference in beta diversity was observed.

The uromycobiome results of these two studies are especially notable in the context of UTI. Bacteria and fungi can both be etiological agents of infection, and recent decades have seen a rise in UTIs caused by fungal species, such as those of genera *Candida* (65, 66). This genus was also observed in the adult cohort of Moustafa et al. (57). While the majority of those with UTI in the study by Forster et al. (29) cultured *E. coli* (92%)*, Candida parapsilosis* differed strongly compared to those without UTI. There have been several reported interactions between *E. coli* and *Candida* that suggest their interplay in promoting infection (67–71). Meanwhile, Wehedy et al. (22) did not detect *Candida* at a similar abundance in their cohort of healthy children. This could further suggest an important link between the composition balance of the uromycobiome and urinary health. More research is needed to confirm this and establish a baseline for the healthy uromycobiome in children.

Wehedy et al. (22) has been the only study, to date, that has examined the urinary virome in children. Caudovirales, Tymovirales, and Herpesvirales were observed to be the most abundant orders, and *T4 Virus, Betapartitivirus, Tymovirus,* and *alpha-partitivirus* were the most abundant genera. Like their uromycobiome analyses, there was no significant difference in beta diversity between the two sexes. However, at the species level, *Mastadenovirus* and the *Human Mastadenovirus-A* virus were notably identified in males compared to females, and *Dill cryptic virus 2* and *Chrysochromulina ericina* virus were in higher abundance in females compared to males. The greatest differences between age groups were observed in virome composition, with an observed increase in abundance with age of order Caudovirales, genus *T4 Virus*, and *Shigella phage SHFML-11*. Compared to the >10 years age group, the 1–5 age group had higher alpha diversity and higher presence of *Herpesvirales, Shigella phage SHFML-11 Rosellinia necatrix partitivirus 2,* and *red clover cryptic virus 2*. The urinary virome has been reviewed for its potential connection with human pathology and health (72), and it has been suggested to be an alternative mode of screening in the context of human viruses, such as human papillomaviruses in adults (73). However, no studies have linked the urinary virome to disease in children. Bacteriophages and other viruses have been detected in adult urine samples (64), but the overall connection between viruses to urinary tract health and urobiome health has not been resolved.

### 3.4 The pediatric urobiome as a biomarker for mental disorders

As previously discussed, the human microbiome has been linked to the health and disease of the host, and extensive work has recently gone into exploring the microbiome as an indicator of shifts between these states (74). reviewed the potential for human microbiomes to be analyzed for biomarkers of a wide variety of diseases and disorders, including those that affect the skin, respiratory system, immune system, and metabolism, as well as in the cases of infection and cancers. Mental disorders such as depression and anxiety have also been included in the research mapping microbiome biomarkers to disease, specifically by linking gut microbiome features to symptoms measured by clinical interviews and other diagnostic criteria, as reviewed by Simpson et al. (75).

Cho et al. (30) has been the only study, to date, to examine the urobiome as a potential biomarker for a mental disorder, specifically for those with attention-deficit hyperactivity disorder (ADHD). 33 males aged 6–15 years diagnosed with ADHD were compared against 39 adult men aged 19–23 years without ADHD. While there were no statistically significant differences in alpha diversity, there were significant differences in abundance and beta diversity between the two groups. Phyla Firmicutes and Actinobacteria were more abundant in CCMSU samples of those with ADHD, while healthy controls exhibited a greater abundance of Proteobacteria. Genera *Ralstonia* and *Afipia* were more abundant in those with ADHD, while *Corynebacterium* and *Peptoniphilus* were more abundant in healthy controls. Genus *Afipia* had strong correlations with two subscale t-scores for diagnosing ADHD, Child Behavior Checklist and DMS-oriented ADHD, but not with the ADHD Rating Scale IV scores, which were identified as being the “current gold standard” for diagnosis. Further study is required to evaluate these findings. This study was limited by its lack of age-matched healthy controls. However, the authors identify their conclusions based on the findings by Kassiri et al. (20) and other adult male urobiome studies that suggest certain stability of the healthy male urobiome across ages.

## 4 Larger gaps to be addressed in future studies

### 4.1 Genomic and functional studies are needed

The implication of the frequently employed 16S rRNA sequencing is that it only provides taxonomic, compositional information about present microbes. This method cannot provide functional genomic information needed for analysis of traits such as virulence and fitness factors and required for understanding urobiome development. While there are now several compositional studies of the pediatric urobiome, with a couple including EQUC isolation of constituents, only one study proceeded with complete genome sequencing and analyses of identified constituents: *Actinotignum schaali* and *Actinotignum sanguinis* in Reasoner et al. (21). In their enhanced culture *Actinotignum* spp. was obtained most often in the culturable urine samples (28.1%). Notably, their initial 16S rRNA results had also filtered out *Actinotignum* ASVs, and MALDI-TOF had identified all nine of their culturable isolates as *A. schaalii*. Further whole genome sequencing of these isolates reclassified five of them as *A. sanguinis*. All nine isolates possessed several potential factors of urinary tract fitness, including genes for enterobactin transport, heme acquisition, and copper detoxification systems. Additionally, they reported two potential virulence factors: *ermX*, which confers macrolide resistance, and Esx-1 Type VII secretion/*esxA*, which is an anti-eukaryotic membrane-permeabilizing toxin found in *Mycobacterium* *tuberculosis* pathogen and gram-positive bacteria. This is of special interest because *A. schaalii* is now being recognized as an opportunistic causative agent of UTI with increasing incidence (21).

To date, no functional studies analyzing behavior or interactions of isolated species from pediatric urobiome were reported in either *in vitro* or *in vivo* models, though there have been a few functional studies of the common bacteria found in the adult urobiomes (76–81). However, the genomes of numerous urobiome commensal strains have been established and analyzed bioinformatically, even with establishing new speciation of bacteria specific to the human bladder (8, 82–87).

### 4.2 Female cohort studies

Due to complications arising from the specifics of the pediatric population, the existing studies either work with both sexes or only male participants; the only exception is the single female-only cohort of Cole et al. (27) examining the urobiome in females with BBD. To date, there are no other female-only studies, and there are no studies at all that examine the healthy urobiome of exclusively females across any age range. While the importance of understanding urobiome development for recurrent UTI studies in female participants is clearly shown (54), no studies have yet addressed this issue carefully in female children.

### 4.3 Replicated cohorts and methods benchmarking

While we have attempted to identify similarities in the makeup of the pediatric urobiome across studies, it is difficult to draw definitive conclusions, mainly because some studies collect data for either very narrow or very broad age ranges (Table 1) and the analyses of changes observed are done in different cohorts. For example, the broader age range of boys in Storm et al. (19) and reported analyses cannot be truly, directly aligned with the narrow age range of boys in Reasoner et al. (21) study. The physical features of these cohorts regarding the health status of the urinary tract do not always match, which further complicates comparisons. We thus reiterate the importance and need for further, replicated studies of the urobiome in a variety of pediatric cohorts to promote a robust understanding of its development and dynamics. Creation of unified and standardized databases for pediatric and adult urobiome studies will be helpful in comparative analyses going forward (82).

Additionally, it is important to critically evaluate and identify gaps in current benchmarking and optimization studies for pediatric urobiome applications. For example, many of the laboratory methods have been evaluated for optimization on adult human and canine urines (88–90), and several comparisons of detection methods (91), and bioinformatics (38, 41) specifically for the urobiome. It is also essential that investigators in the pediatric urobiome research community employ current recommendations developed by the urobiome (15), kidney microbiome (92), and general microbiome communities (93) and develop consensus on standards and guidelines for the field.

## 5 Conclusions

The knowledge base of the urobiome is still not as robust as that of more extensively studied human microbiome niches. Nonetheless, the present urobiome research has hit notable milestones in studying the human microbiome, and common patterns in makeup and abundance across cohorts begin to present themselves. While sampling pediatric populations remains a unique challenge, advancements in urobiome sequencing have improved the ability of researchers to discern greater detail regarding urobiome composition and dynamics, especially in regard to the immense diversity of microbial species yet to be explored in greater detail. As the field of urobiome research enters its second decade, a more detailed study of the pediatric urobiome will be vital in understanding the crosstalk between urinary microbiota and a lifetime of urinary health and disease.
